# Prevalence and associated factors of hypertension among adult patients in Felege-Hiwot Comprehensive Referral Hospitals, northwest, Ethiopia: a cross-sectional study

**DOI:** 10.1186/s13104-018-3986-1

**Published:** 2018-12-10

**Authors:** Amare Belachew, Tilahun Tewabe, Yihun Miskir, Ermias Melese, Endalew Wubet, Saba Alemu, Tewabech Teshome, Yezibalem Minichel, Getasew Tesfa

**Affiliations:** College of Medicine and Health Science, Bahir Dar University, Bahir Dar, Ethiopia

**Keywords:** Hypertension, Prevalence, Determinants, Northwest Ethiopia

## Abstract

**Objective:**

The main objective of this study was to assess the prevalence and factors associated with hypertension among adult patients in Felege-Hiwot Comprehensive Referral Hospital, northwest Ethiopia, 2018.

**Result:**

The prevalence of hypertension in the current study area was 27.3%. Known history of cardiac problems [AOR = 6.9; 95% CI (1.24, 11.44)], alcohol consumption [AOR = 2.2; 95% CI (1.04, 5.05)], abdominal obesity [AOR = 2.3; 95% CI (1.02, 5.04)], and obesity [AOR = 4.8; 95% CI (1.12, 8.34)] were factors associated independently with hypertension.

## Introduction

Hypertension (HTN) is defined as a persistent raised systolic or diastolic blood pressure equal to or more than 140/90 mmHg in adults aged 18 years and over [1, 2]. It usually associated with other chronic non-communicable diseases such as myocardial infarction, congestive heart failure, stroke and chronic kidney diseases (CKD) [1, 3–5].

High blood pressure becomes a trait as opposed to a specific disease and represents a quantitative rather than a qualitative deviation from the norm [3]. Level of hypertension varies among countries and apparently prevalent in developed countries and urban populations due to lifestyle changes associated with civilization [1]. Currently, Both developed as well as developing countries are at increased risk of developing hypertension [1, 6].

According to world health organization report of 2009, worldwide prevalence of hypertension in adults aged 25 years and above was 40%, highest in Africa 46% and lowest in America (35%) [1]. Another report showed that in 2010 the world wide prevalence of hypertension was more than 1.3 billion individuals, represents 31% of adults [7]. Hypertension is estimated to cause 7.5 million deaths annually, accounting for 57 million disability-adjusted life years [8]. It is a risk factor for the development of cardiovascular disease and stroke [9]. Studies in Ethiopia showed that the prevalence of hypertension both in rural and urban inhabitants. It ranges from 13 to 30% [10–15].

The prevention and control of hypertension have not received due attention from many developing countries [10]. However, awareness about treatment and control of hypertension is extremely low among developing nations including Ethiopia [16]. Therefore, the purpose of this study was to assess the prevalence and factors associated with hypertension among adult patients who visited the outpatient department in Felege-Hiwot Comprehensive Referral Hospitals, northwest, Ethiopia.

## Main texts

### Method

#### Study area and participants

An institutional based cross sectional study was conducted at Felege-Hiwot Comprehensive Referral Hospital from March 12 to May 2, 2018. This Hospital is found in Bahir Dar, the Capital City of the Amhara regional state and 565 km away from Addis Ababa, the Capital City of Ethiopia. Felege-Hiwot Comprehensive Referral Hospital is a tertiary referral hospital with 400 beds capacity and around 15 adult outpatient department (OPD) serving over 7 million people from the surrounding area. The OPDs serves around 900 patients per day. The hospital provides obstetrics, pediatrics, internal medicine, ophthalmology, general, gynecology, ENT (ear, nose, and throat) and orthopedic surgery services. A wide range of procedures are performed electively and the emergency caseload is high with a large volume of trauma.

#### Sample size determination and sampling techniques

The required sample size was determined using a single population proportion formula by considering the following assumptions; prevalence (P) = study conducted in northwest Ethiopia on June 2014, the overall prevalence of hypertension was found to be 24% [2], confidence level (CL) = 95%, 5% degree of precision, add non-response rate of 10% and the final sample size was 308. There were 19 adult OPDs in FHRH. Among them, 10 OPDs were selected by lottery method. Systematic sampling technique was used to select study participants. After the first respondent drawn by lottery method every 3 patients were interviewed and then consquative sampling was employed until the sample size was reached.

#### Data collection techniques and procedures

A structured interview administered questionnaire was used to collect the data related with socio-demographic characteristics and check list was used to collect data from physical examination findings such as body weight, height, hip and waist circumferences, and three BP records were used for data collection. Blood pressure was measured by using adult size mercury sphygmomanometer and stethoscope. Each individual patient’s BP was taken while the patient was in a sitting position, from the right arm after the patient rested for at least 5 min before measurement. Consumption of caffeinated products such as coffee, Coca-Cola or tea was assed carefully and BP was taken by considering the time effect (after 30 min of initial consumption). Additionally, activities such as smoking and exercising were also been avoided 30 min prior to measure of BP. Three measurements of BP on a single visit were taken at least 3 min apart, and the average of the three records was used for the computation of results.

#### Data analysis

Data were coded and entered into Epi info version 3.5.1 and transferred into SPSS version 20 for analysis. Descriptive statistics like frequency table was used to present the results. Both Bivariate and multivariate analyses were used. Variables with a p-value of less than 0.05 from the Bivariate were finally adjusted into multivariate analysis. Variables with a p-value < 0.05 were considered as significant predictors of hypertension.

#### Operational definitions

*Hypertension* Hypertension was defined as having Systolic BP ≥ 140 mmHg or Diastolic BP ≥ 90 mmHg or reported use of regular anti-hypertensive medications prescribed by professionals for raised BP [2].

*Positive smoking history* Based on patients’ history of using manufactured or locally-made tobacco.

*Alcohol use* Refers to the consumption of local or manufactured alcohol beverages on a daily basis.

*Regular chat chewers* Individuals who reported chat use for 5 days or more in a week, and this was considered to be clinically significant.

*Overweight* Body mass index (BMI) ≥ 25 but less than 30 kg/m^2.^

*Obese* Patients were declared obese when their BMI being above 30 kg/m^2^.

*Abdominal obesity* Defined as waist-to-hip ratio (WHR) greater than 0.85 m for women and above 1 m for men.

### Result

#### Socio-demographic characteristics of participants

Out of the total patients who were attending OPD; 50.3% were males, 45.8% were in between 18 and 49 age groups, 64.6% were orthodox by religion, 58.1% were married, 48.7% attended grade 9 and above, 47.7% were governmental employers and around 32.6% of them earned more than 300 ETB per month (Table 1).

**Table 1:** Socio demographic characteristics of respondents (adult clients) see in Felege Hiwot Comprehensive Referral Hospitals, Bahir Dar, Ethiopia, 2018

Variables	Frequency	Percent (%)
Age		
18–49	138	44.8
50–65	107	34.7
> 65	63	20.5
Sex		
M	153	49.7
F	155	50.3
Marital status		
Single	47	15.3
Married	179	58.1
Divorced	45	14.6
Widowed	37	12
Illiterate	42	13.6
Read and write	91	29.6
Primary	25	8.1
Higher	150	48.7
Occupation		
Farmer	45	14.6
Merchant	55	17.9
Govt employee	147	47.7
House wife	24	7.8
Student	30	9.7
Soldier	7	2.3
Religion		
Orthodox	199	64.6
Protestant	17	5.5
Muslim	69	22.4
Catholic	23	7.5
Monthly income		
< 1000	37	12
1001–2000	68	22
2001–3000	103	33.4
3001–4000	66	21.5
4001–5000	34	11.1

Among the study participants, 27.3% had hypertension, 2.3% had known history of cardiac and related diseases, 21% had a previous history of hypertension and 3.2% were cigarette smokers. Out of all, 12% were drank alcohol frequently and 47.7% had the optimal recording of blood pressure. Anthropometrics measurement of participants; base on waist and hip circumferences around 9% of females and 5.3% of males had a high risk to develop hypertension (Table 2).

**Table 2:** Hypertension and its risk factors among respondents in adult clients visit outpatient department FHRH, Bahr Dar, Ethiopia, 2018

Variables	Frequency	Percent (%)
Family history of hypertension		
Yes	28	9
No	280	91
History of CVDs		
Yes	7	2.3
No	301	97.7
History of renal disease		
Yes	0	0
No	308	100
History of DM		
Yes	5	1.6
No	303	98.4
Previous history of HTN		
Yes	64	20.6
No	372	79.4
Repeat hypertensive patients that had follow up (64)		
Yes	60	93.8
No	4	6.2
Started antihypertensive treatment		
Yes	60	93.8
No	4	6.2
Cigarette smokers		
Yes	10	3.2
No	298	96.7
Alcohol users		
Yes	37	12
No	271	88
Frequency of alcohol taking (37)		
1–2 per days	8	21.6
3–4 per days	10	27.1
5–6 per days	15	40.5
Every days	4	10.8
Label of physical exercise		
High or vigorous	36	11.7
Medium	97	31.5
Low	175	56.8
Blood pressure		
< 120/80	147	47.7
120–130/80–85	55	17.9
130–139/85–89	22	7.1
140–159/90–99	41	13.3
160–179/100–109	31	10.0
> 179/109	12	4
Hip circumference of men (153) (cm)		
< 93	150	98.1
≥ 94	3	1.9
Waist circumference of men (153)		
< 101	134	87.5
≥ 102 cm	19	12.5
Waist to hip ratio of men (153)		
≤ 0.9 cm	118	77.2
0.91–0.99	25	16.3
≥ 1	10	6.5
Hip circumference of women (155)		
< 79	150	96.8
≥ 80 cm	5	3.2
Waist circumference for women (155) (cm)		
< 87	135	87.1
≥ 88	20	12.9
Waist to hip ratio of women (155)		
≤ 0.8	101	65.2
0.81–0.85	40	25.8
≥ 86	14	9
BMI		
Under weight	32	10.4
Normal	246	79.9
Overweight	17	5.5
Obese	13	4.2
Diet		
Vegetarian	56	18.2
Non-vegetarian	120	39
Ovo-vegetarian	132	42.8
Abdominal obesity		
Yes	24	7.8
No	284	92.2

#### Factors associated with hypertension

In bivariate analysis: age, sex, Alcohol taking, BMI, waist to hip ratio and history of CVDs were found to be significantly associated factors of Hypertension.

Finally, drink alcohol, BMI and waist to hip ratio were identified as predictor variables in the multivariate analysis method.

Among all, those who have known cardiac cases were 6.9 times more likely to be hypertensive when compared to those who haven’t [AOR = 6.92 (1.246, 11.44)].

Central obesity measured with waist circumference and BMI were major modifiable risk factors to develop hypertension. Obese individuals had more than 4.79 fold risk of being hypertensive in comparison to underweight subjects in this study [AOR = 4.79 (1.129, 8.349)].

Abdominal obesity was the main risk factor to develop hypertension. Individuals having an abnormal waist to hip ratio were 2.3 folds risk of being to develop hypertension in comparisons to normal waist to hip ratio [AOR = 2.3 (1.025, 5.043)].

Alcohol consumption was the major risk factor in the developed countries to develop hypertension. Hypertension was more prevalent in alcohol users [AOR = 2.22 (1.045, 5.059)] (Table 3).

**Table 3:** Factors that affect Hypertension among adult client visit OPD in bivariate and multivariate logistic regression analysis model in Felege Hiwot Comprehensive Referral Hospital, Bahir Dar, Ethiopia, 2018

Variables	Hypertension			
	Yes (N & %)	No (N & %)	COR (95% CL)	AOR (95% CL)
Age				
18–49	30 (21.7)	108 (78.3)	1	
50–65	32 (29.9)	75 (70.1)	1.536 (.861, 2.74)	
> 65	22 (34.9)	41 (65.1)	1.932 (1.001, 3.727)	
Sex				
M	50 (32.7)	103 (67.3)	1.728 (1.039, 2.873)	
F	34 (21.9)	121 (78.1)	1	
History of cardiac disease				
Yes	5 (71.4)	2 (28.6)	7.025 (1.336, 36.941)	6.92 (1.246, 11.44)*
No	79 (26.2)	222 (73.8)	1	
Drink alcohol				
Yes	19 (51.4)	18 (48.6)	3.345 (1.657, 6.753)	2.22 (1.045, 5.059)*
No	65 (24)	206 (76)	1	
BMI				
Under-weight	8 (25)	24 (75)	1	
Normal	60 (24.4)	186 (75.6)	0.968 (.413, 2.267)	
Overweight	8 (47.1)	9 (52.9)	2.667 (.769, 9.251)	
Obese	8 (61.5)	5 (38.5)	4.8 (1.214, 18.97)	4.79 (1.129, 8.349)*
Central obesity				
Non obese	112	172	1	
Obese	16	8	3.07 (1.755, 4.89)	*2.3 (1.025, 5.043)*

Significant values are in italics

1 = Reference, * p-value less than 0.05, *N* number, % percent

### Discussion

The prevalence of hypertension in this study was 27.3% which is comparable to studies done in Gonder 27.9% [10], Uganda 27.2% [17] and Jigjiga-Somali Ethiopia 28.3% [12]. This finding is lower than studies done in Bayelsa State 50.4% [18], Brazil 29.5% [19], Nigeria 33.1% [20], Hossana-Hadiya zone 30% [11], Urban Varanasi 32.9% [21]. However, this finding is higher than studies done in Bedele town 22.4% [13], Durame town 22.4% [15] and southern Ethiopia 13.2% [14]. This may be due to; the difference in socio-demographic characteristics like age, income, education, resident etc.; the difference in study settings, the habit of visit of health setups and dietary intakes.

Co-morbid diseases are risk factors for hypertension. In this study, participants with known history of cardiac diseases were 6.9 times more likely to develop hypertension than their counterparts. This finding is in line with studies done in Jijiga [12], Brazil [19] and Jimma [22] where being a known cardiac patients was reported as a risk factor for developing hypertension [23].

Alcohol consumption is a third major risk factor to develop hypertension. Alcohol users were 2.2 times more likely to develop hypertension than nonusers. This finding is similar to studies done in Gonder [10], Brazil [19], systematic review studies in Columbia [24]. The possible explanation is alcohol increases stimulation of sympathetic nervous system, endothelin, insulin resistance and inhibition of vascular relaxing substances which leads to hypertension. According to the general facts of alcohol and hypertension states; regularly drinking alcohol increase substantially the risk of developing hypertension and up to 75% of individuals develop hypertension when taking alcohol more than 3 times a day. Therefore, persons should reduce alcohol intake especially for those risk groups like cardiac problems, liver problems, and other chronic co-morbid diseases.

Peoples of BMI > 30 were 4.79-folds more likely to develop hypertensive than underweight individuals. This finding is consistent to studies done in Gonder [10], Jijiga [12], Bayisa state [18], Nigeria [20], Urban Varanasi [21], Jimma [22] and Kenya [23]. The possible explanation is when a person is obese he/she has excess bad cholesterol in blood vessels compared to underweight people and it makes narrow blood vessels and progressively the person develop hypertension due to hormonal effects. Epidemiological studies showed that BMI is the independent predictor for hypertensions [23]. Sodium retention, plasma rennin activity, angiotensinogen, angiotensin II and aldosterone values display significant increase during obesity and additionally insulin resistance and inflammation may promote an altered profile of vascular function and consequently leads to hypertension.

Peoples of abnormal waist to hip ratio were 2.3 times more likely to develop hypertension in compared to for those having the normal ratio. This finding is concurrent with studies done in Bayisa state [18], Urban Varanasi [21], Bedele town [13], Jimma [22] and Kenya [25]. The possible explanation is; waist for hip ratio is the major clinical assessment of central obesity and individuals having abdominal obesity indicate more bad cholesterol in blood vessels and the presence of this fat in blood vessel makes blood vessels narrow and it is the risk for hypertension than compared to persons having the normal ratio. Additionally, obesity increases activation of the sympathetic nervous system, which appears to be mediated in part by increased levels of the adipocyte-derived hormone, leptin, stimulation of pro-opiomelanocortin neurons, and subsequent activation of central nervous system melanocortin 4 receptors and finally results in hypertension.

### Conclusion

In the current study, almost one-third of the study population was hypertensive which mean there was a hidden epidemic in this population. Known history of cardiac diseases, obesity, abnormal waist to hip ratio and alcohol consumption were determinant predictors of hypertension. Efficient health screening and regular checkups, promote healthy lifestyles, minimize alcohol intake and health promotion regarding hypertension should be provided in the population as means of primary prevention.

## Limitation

The study design and setting were the limitation of this study. As it was an institution based cross-sectional study, the overall prevalence of hypertension might be overestimated and it cause-effect relationship did not explained.

## Abbreviations

AOR: adjusted odds ratio
BP: blood pressure
BMI: body mass index
CKD: chronic kidney disease
CL: confidence level
CVD: cardio vascular disease
DBP: diastolic blood pressure
ENT: ear nose throat
ETB: Ethiopian Birr
FHCRH: Felege Hiwot Comprehensives Referral Hospital
HTN: hypertension
OPD: out patient department
SBP: systolic blood pressure
WHO: World Health Organization
WHR: waist to hip ratio
